# Supplementary material for: Dynamics of morphogen source formation in a growing tissue
Source: PLoS Comput Biol. 2024 Oct 14;20(10):e1012508. doi: 10.1371/journal.pcbi.1012508 (PMC11501038; doi:10.1371/journal.pcbi.1012508)
Supplement: S1 Fig — (PDF) [file pcbi.1012508.s001.pdf]

Figure S1

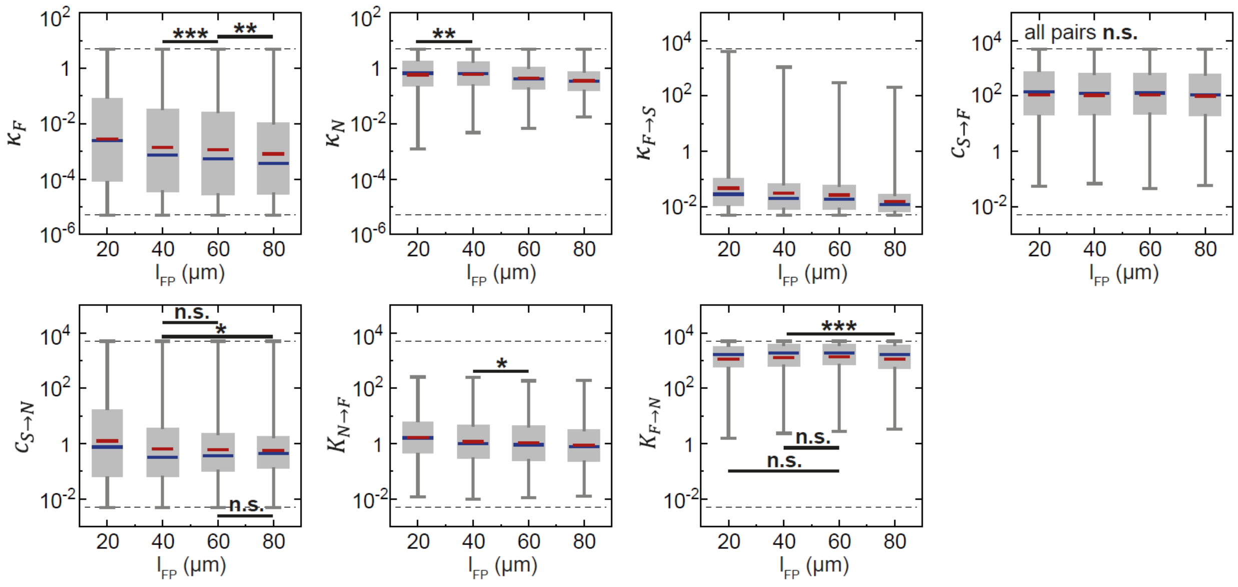

Figure S1. Dependence of FP size on model parameters. Distribution of model parameters for networks that yield the indicated final FP sizes at the end of the simulation (L_{end} = 400 µm at t = 60 h). The boundaries of the allowed range of parameters in the screen are indicated with dashed lines. Box-whisker plots: 25–75 percentile (box), median (blue line), mean (red line), highest/lowest observation (fence). Pairwise comparisons two-tailed t-test: ns, not significant, $P > 0.05$; * $0.05 \geq P > 0.01$; ** $0.01 \geq P > 0.001$; *** $0.001 \geq P > 0.0001$; **** $0.0001 \leq P$. All pairwise comparisons that are not indicated are ****. Sample sizes (number of solutions for given l_{FP}): 20 µm (n = 23116), 40 µm (n = 12832), 60 µm (n = 5254), 80 µm (n = 1772).
