# Supplementary material for: Dynamics of morphogen source formation in a growing tissue
Source: PLoS Comput Biol. 2024 Oct 14;20(10):e1012508. doi: 10.1371/journal.pcbi.1012508 (PMC11501038; doi:10.1371/journal.pcbi.1012508)
Supplement: S2 Fig — (PDF) [file pcbi.1012508.s002.pdf]

Figure S2

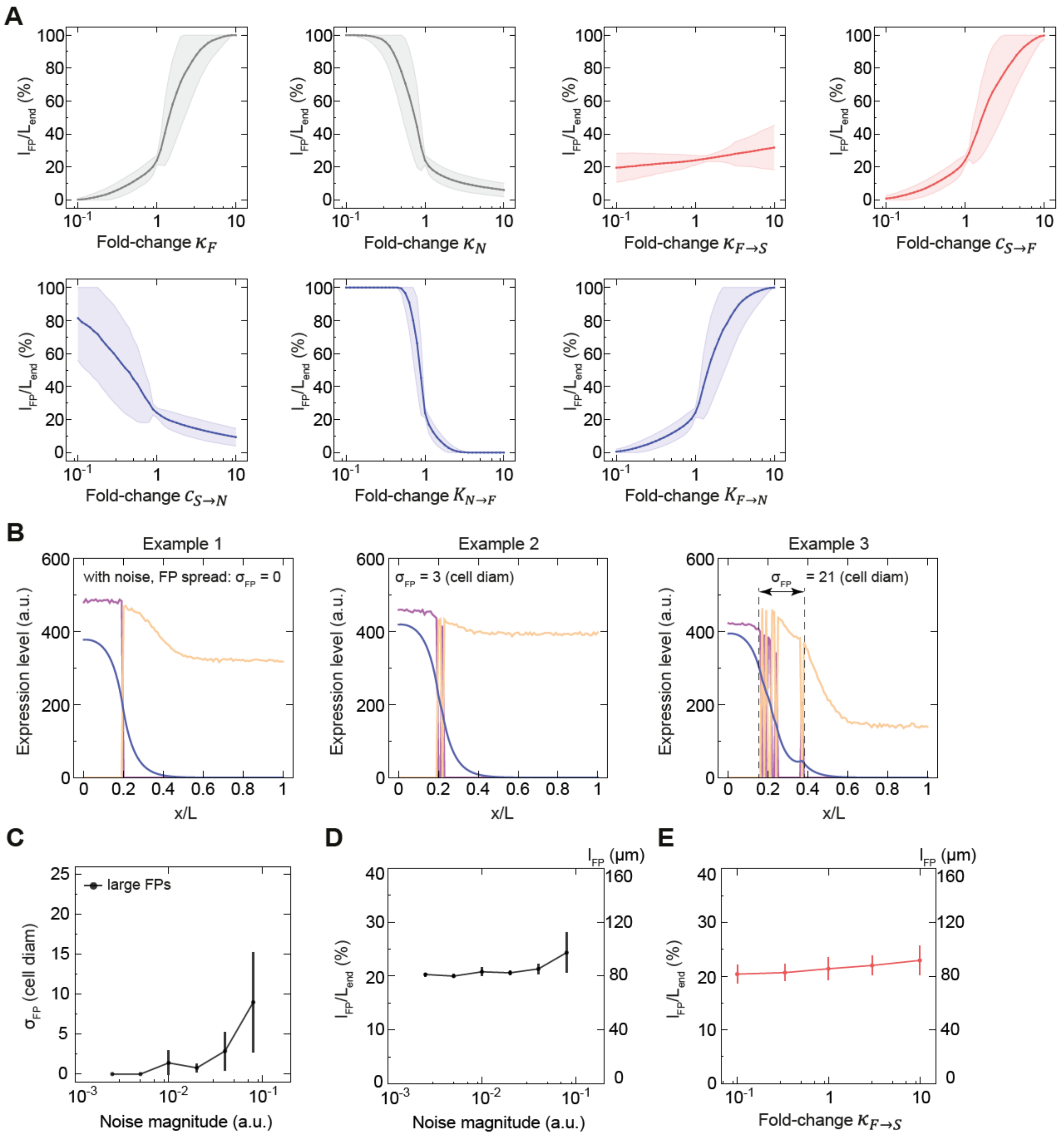

Figure S2. Model results are robust to changes in diffusion and noise in gene expression. A. Sensitivity to parameters (analogous to Fig 2) of the model with increased diffusion of Shh, D_S = 0.22 μm^2 s^{-1}. Mean relative FP size at t = 60 h upon perturbation of the indicated model parameter for a subset of 214 randomly selected networks with large FPs (l_{FP}/L_{end} = 20%). Parameters are modified in 40 equally distributed logarithmic steps from 0.1 to 10-fold of their value. Shaded regions, SE. **B.** Three examples of gene expression pattern at t = 60 h in simulations with noise (Methods). Parameters sets for large FPs are shown. The noise magnitude is $\sigma_{[F]}$ = $\sigma_{[N]}$ = 0.04 a. u., and correlation time $\tau_{[F]}$ = $\tau_{[N]}$ = 50 s. The FP spread σ_{FP} is defined as a difference between the lowest position of N and the highest position of F in relative units, x/L. The tissue is simulated with 100 bins, with one bin, corresponding to σ_{FP} = 0.01 and one cell diameter. For the selected magnitude of noise, most insensitive solutions exhibit no change in boundary imprecision with σ_{FP} = 0 (60% of solutions, example 1), moderate change $\sigma_{FP} \leq 0.05$ (20%, example 2, consistent with (Zagorski et al., 2017, [18] in main text)), and large change 0.05 < σ_{FP} ≤ 0.25 (10%, example 3). We exclude cases with σ_{FP} > 0.5 (10%). The

mean (± SEM) FP spread is σ_{FP} = 0.03 ± 0.02. The FP size l_{FP}^{noise} defined as the mid position between the lowest position of N and the highest position of F is l_{FP}^{noise} = 0.21 ± 0.01 (mean ± SEM), n = 10. **C.** FP spread σ_{FP} for varied gene expression noise. The noise is varied from $\sigma_{[F]}$ = $\sigma_{[N]}$ = 0.0025 a.u., by doubling the magnitude, up to $\sigma_{[F]}$ = $\sigma_{[N]}$ = 0.08 a. u. Error bars SEM, n = 10. **D.** Relative FP size for varied noise. Noise up to $\sigma_{[F]}$ = $\sigma_{[N]}$ = 0.04 a.u. only weakly affects the results. Error bars SEM, n = 10. **E.** Change in mean FP size for fixed noise $\sigma_{[F]}$ = $\sigma_{[N]}$ = 0.04 a.u. and varied $\kappa_{F\to S}$. The FP size follows the same trend as in A (fold change of $\kappa_{F\to S}$) indicating that noise does not affect the insensitivity of FP size to Shh production in the FP. Error bars SE, n = 10.
