# Supplementary material for: Dynamics of morphogen source formation in a growing tissue
Source: PLoS Comput Biol. 2024 Oct 14;20(10):e1012508. doi: 10.1371/journal.pcbi.1012508 (PMC11501038; doi:10.1371/journal.pcbi.1012508)
Supplement: S3 Fig — (PDF) [file pcbi.1012508.s003.pdf]

Figure S3

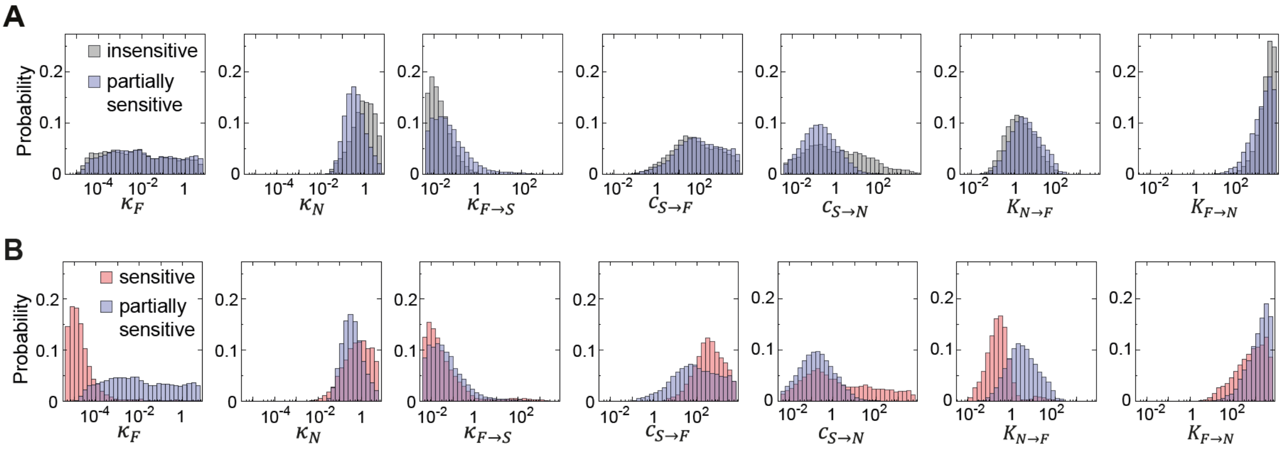

Figure S3. Model parameters for different mechanisms of FP formation. A. Distribution of model parameters for Shh[FP]-partially sensitive (blue) and insensitive (grey) solutions. In insensitive solutions the FP size does not change when Shh[FP] is removed ($\kappa_{F\to S}$ = 0), whereas in partially sensitive solutions the FP size is decreased when Shh[FP] is removed. **B.** The same as A, but for Shh[FP]-sensitive (red) and partially sensitive (blue) solutions. For sensitive solutions, the FP is completely lost when Shh[FP] is removed. A, B, insensitive (n = 66 827), partially sensitive (n = 55 911), sensitive (n = 45 336).
