# Supplementary material for: Dynamics of morphogen source formation in a growing tissue
Source: PLoS Comput Biol. 2024 Oct 14;20(10):e1012508. doi: 10.1371/journal.pcbi.1012508 (PMC11501038; doi:10.1371/journal.pcbi.1012508)
Supplement: S4 Fig — (PDF) [file pcbi.1012508.s004.pdf]

Figure S4

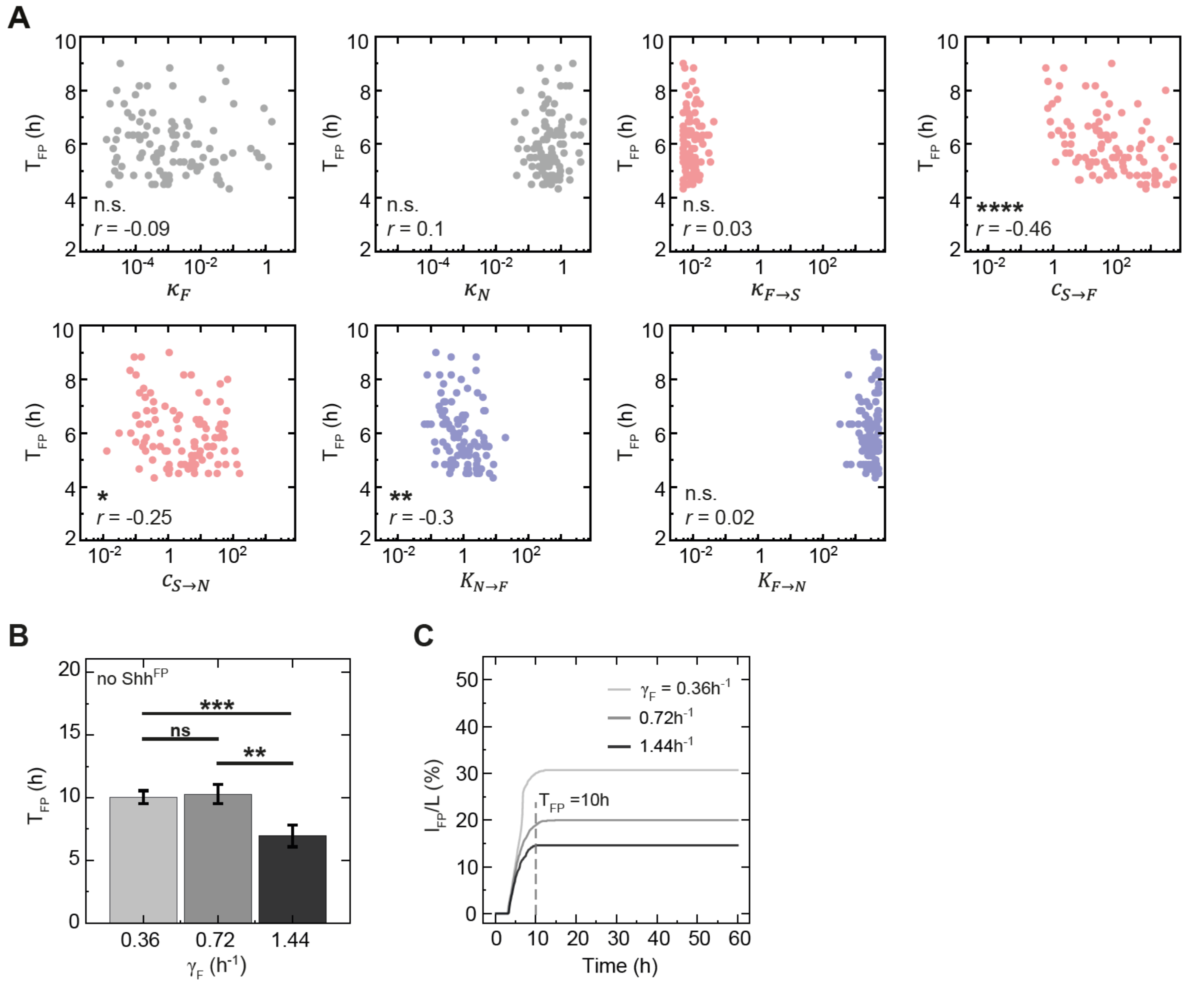

Figure S4. Dependence of FP formation time on model parameters. A. FP formation time T_{FP} for insensitive solutions upon perturbation of model parameters. T_{FP} is the time at which the FP reaches its final relative FP size. Pearson's correlation coefficient *r* between T_{FP} and log of parameter is reported. Pearson correlation test *P*-values: ns, not significant, $P > 0.05$; * $0.05 \geq P > 0.01$; ** $0.01 \geq P > 0.001$; *** $0.001 \geq P > 0.0001$; **** $0.0001 \leq P$. n = 100 solutions per parameter. **B.** Mean T_{FP} as a function of the degradation rate of F (γ_F), see Eq. 1. Pairwise comparisons two-tailed *t*-test, significance levels as in A. n = 10 per condition, error bars SEM. **C.** Relative FP size as a function of time for different γ_F. The default condition is γ_F = 0.72h^{-1}, dashed line indicates the position of the average T_{FP} = 10 h for that condition.
