# Supplementary material for: Dynamics of morphogen source formation in a growing tissue
Source: PLoS Comput Biol. 2024 Oct 14;20(10):e1012508. doi: 10.1371/journal.pcbi.1012508 (PMC11501038; doi:10.1371/journal.pcbi.1012508)
Supplement: S5 Fig — (PDF) [file pcbi.1012508.s005.pdf]

Figure S5

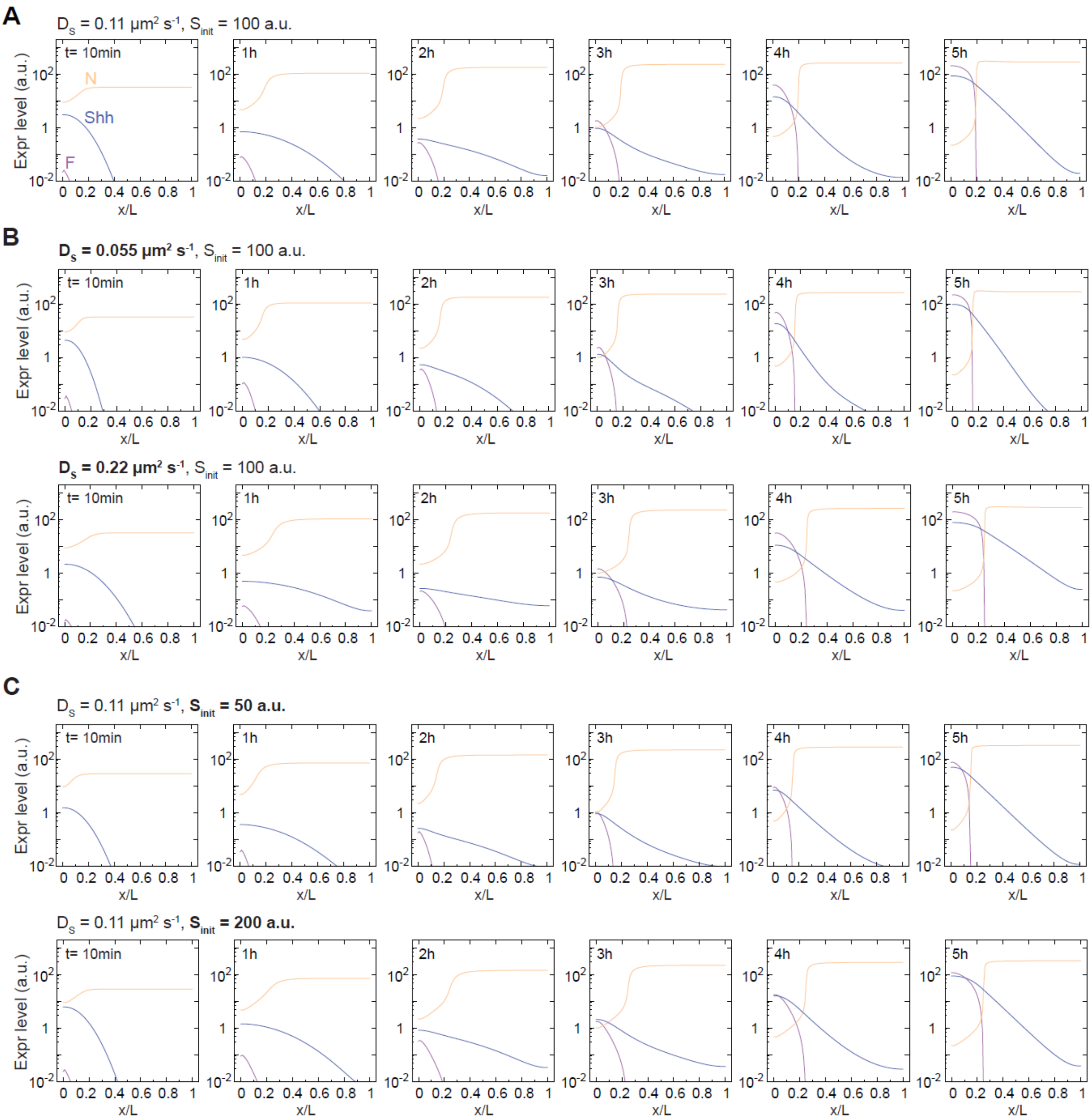

Figure S5. Initial dynamics of the Shh gradient and FP formation. A. Temporal dynamics of Shh and gene expression profiles at early times, prior to 5h. Floor plate F (purple), neural progenitor domains N (yellow), Shh (blue). The default parameters are used with D_S = 0.11 µm^2 s^{-1} and initial burst condition S_{init} = 100 a.u. **B.** Same as A, but with a two-fold reduction (top row) or increase (bottom row) of the diffusion coefficient. **C.** Same as B, but with a two-fold reduction (top row) or increase (bottom row) of S_{init}. In A-C the same parameter set was used except for the indicated changes.
