# Supplementary material for: Dynamics of morphogen source formation in a growing tissue
Source: PLoS Comput Biol. 2024 Oct 14;20(10):e1012508. doi: 10.1371/journal.pcbi.1012508 (PMC11501038; doi:10.1371/journal.pcbi.1012508)
Supplement: S6 Fig — (PDF) [file pcbi.1012508.s006.pdf]

Figure S6

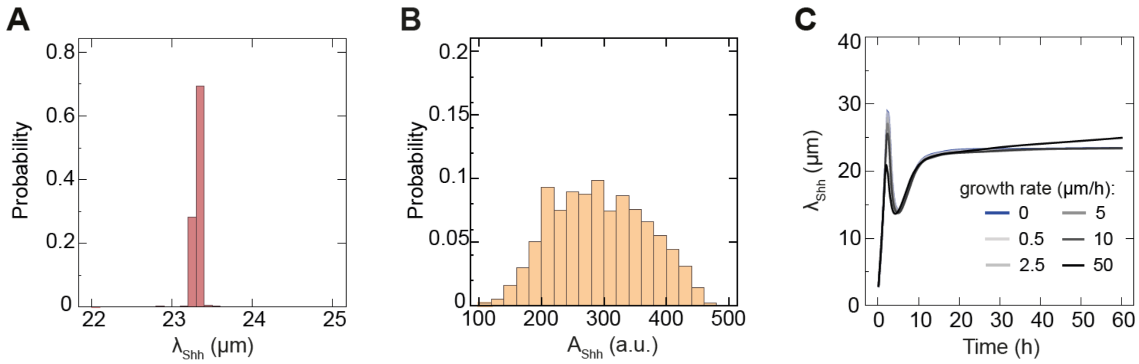

Figure S6. Shh decay length and amplitude in the computational screen. A. Probability distribution of Shh decay lengths in successful solutions of the computational screen. **B.** Probability distribution of Shh amplitudes. A, B, n = 169 979. **C.** Shh decay length for Shh^{FP}-insensitive solutions as a function of time for different growth rates from k_p= 0 µm/h to 50 µm/h (color coded as in the legend). The Shh decay length deviates from the value predicted by λ_{Shh} = $\sqrt{D_S/\gamma_S}$ = 23.5 µm only for extremely fast growth. Sample size, n = 10 per condition, sampled every 10 min.
