# Supplementary material for: Dynamics of morphogen source formation in a growing tissue
Source: PLoS Comput Biol. 2024 Oct 14;20(10):e1012508. doi: 10.1371/journal.pcbi.1012508 (PMC11501038; doi:10.1371/journal.pcbi.1012508)
Supplement: S7 Fig — (PDF) [file pcbi.1012508.s007.pdf]

Figure S7

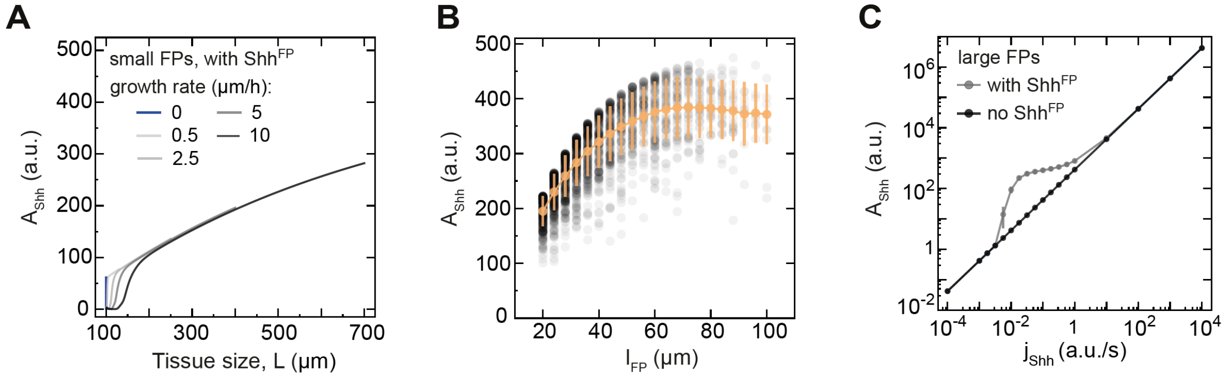

Figure S7. The Shh amplitude depends on the tissue size and Shh flux. A. Shh amplitude as a function of tissue size for insensitive solutions with Shh^{FP} resulting in small FPs (l_{FP}/L_{end} = 5%). Growth rates from k_p = 0 μm/h to 10 μm/h are color-coded, n = 10 per condition, sampled every 10 min. **B.** Shh amplitude as a function of FP size throughout time. The yellow points correspond to mean A_{Shh} for a given l_{FP}, the number of samples per point from n = 23 116 (l_{FP} = 20 μm) to n = 497 (l_{FP} = 100 μm), error bars SE. Randomly selected points from successful solutions (black), n = 3000. **C.** Shh amplitude for large FP (l_{FP}/L_{end} = 20%) solutions with varied flux of Shh and no initial pulse of Shh. Without Shh^{FP} (black), the A_{Shh} increases linearly with j_{Shh}. In solutions with Shh^{FP} present (grey), the amplitude is increased in the range of ~0.01 ≤ j_{Shh} ≤ ~1 a.u./s. The datapoints for the condition with Shh^{FP} are the same as in Fig 6D (flux present condition). Sample size, n = 10 per condition, error bars SEM. For the condition with no Shh^{FP}, the sampled points are identical for a given flux.
