# Supplementary material for: Dynamics of morphogen source formation in a growing tissue
Source: PLoS Comput Biol. 2024 Oct 14;20(10):e1012508. doi: 10.1371/journal.pcbi.1012508 (PMC11501038; doi:10.1371/journal.pcbi.1012508)
Supplement: S8 Fig — (PDF) [file pcbi.1012508.s008.pdf]

Figure S8

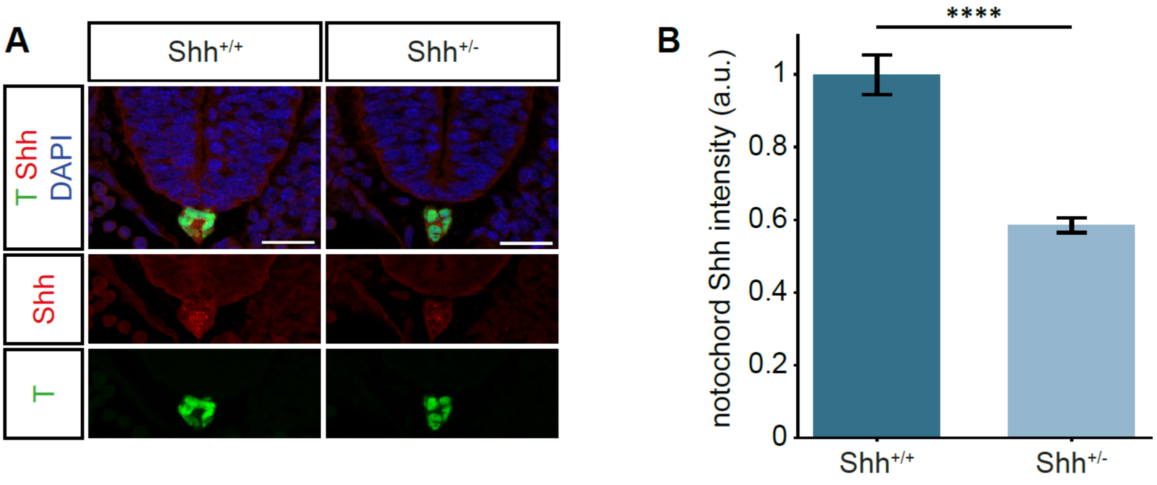

Figure S8. Shh levels in the notochord are reduced in Shh heterozygous embryos at E8.75. A. Representative mouse brachial sections of E8.75 wildtype and $Shh^{+/-}$ littermates. Immunostaining as indicated. Scale bar = 30 µm. **B.** Quantification of the Shh intensity in the notochord in the experiment in A. Two-tailed *t*-test: $P < 0.0001$. Error bars show SEM. Number of sections: n = 59 (control), n = 54 (mutant).
