# Supplementary material for: Dynamics of morphogen source formation in a growing tissue
Source: PLoS Comput Biol. 2024 Oct 14;20(10):e1012508. doi: 10.1371/journal.pcbi.1012508 (PMC11501038; doi:10.1371/journal.pcbi.1012508)
Supplement: Attachment — Submitted filename: reply_to_reviewers_PLCB_Ho_et_al.pdf [file pcbi.1012508.s014.pdf]

Comments to the Authors:

Reviewer #1:

I have read through the manuscript "Dynamics of morphogen source formation in a growing tissue" by R D J G Ho, K Kishi, M Majka, A Kicheva and M Zagorski, submitted for possible publication in the journal PLoS Computational Biology.

The manuscript considers the process by which a dynamic source of morphogen, specifically that of Sonic hedgehog (Shh), which the authors have not written in its full form the first time the acronym occurs - which they should have done) emerges in the vertebrate neural tube as it grows during development using mathematical modeling - whose results they confirm via experiments. Given that my background is that of a theorist, I am unable to comment on the experimental aspects of the paper and will focus solely on the mathematical modeling.

The model described in the manuscript primarily considers one of two sources of Shh, namely the floorplate (the other source being the neighboring notochord) a specialized structure that spans the anteroposterior axis of the embryo - it is seen to be conserved among vertebrates. What makes the question interesting is that induction of the floor plate during embryogenesis is itself mediated by Shh. Thus, Shh concentration gradient is formed by a feedback process in that it regulates the growth of its own source (or at least one of them).

The present manuscript looks at the question of how the process of tissue growth during which domain size increases monotonically affects this complex interplay of Shh and its source.

In particular, the authors try to disentangle the relative contributions of the notochord and the floorplate in the growth of Shh concentration.

The manuscript concludes that the growth of the floorplate is initially guided by Shh presumably produced by the notochord, but in later stages is almost solely governed by domain size increase due to tissue growth. Overall, I really like the manuscript and feel that the model results are plausible. I support publication after the authors have considered the following points in a revised version.

We are pleased that the reviewer likes our manuscript and supports publication and we thank them for their positive feedback. We have now written Sonic hedgehog in its full form where it first appears in the abstract and in the main text – we thank the reviewer for noting this.

The model considerably simplifies the complexity of the actual situation by focusing only on three dynamical variables, namely, the 2 mutually repressing factors that lead cells to choose floorplate or neural progenitor fates and the diffusing Shh morphogen. The spatial aspect is simplified to a 1-dimensional array of 100 components. As the domain is assumed to grow linearly over the duration of interest, this tissue growth is numerically implemented by just increasing the length of each of the 100 components. I found this choice to be rather strange, given that the actual growth may also be through increase in the number of cells.

I would therefore request the authors to consider the alternative possibility of increasing the number of components (or "bins" as they refer to them). I understand that this will raise complicated issues of choosing the states of the newly emerged daughter cells but presumably the contents of the "mother bin" can be divided amongst its "daughter bins".

I am also curious as to the effect of nonlinear growth on the results of the paper. I understand that the duration between successive cell divisions can alter quite significantly during development. As a result, the conclusion that the authors reach that Shh produced by the floorplate does not substantially affect its growth could possibly be an artefact of the linear growth rate assumption. It would be nice if the authors check out these two suggestions.

The goal of our approach was to develop the simplest possible model that captures the essential features of the interplay between Shh, the FP and tissue growth. Our implementation of the growth rate is based on previous data indicating that the tissue increases in dorsoventral length from 100μm to 400μm in approximately 60h (Kicheva et al, Science, 2014, DOI: 10.1126/science.1254927; Zagorski et al, Science, 2017, DOI: 10.1126/science.aam5887; Cohen et al, Nat Commun, 2015, DOI: 10.1038/ncomms7709). Indeed, due to the lengthening of the cell cycle over time and increasing cell loss due to terminal differentiation, this growth results from a net growth rate which decreases over time (Kicheva et al, Science, 2014). Because of this, the tissue has been found to exhibit a shallow exponential growth over time (L=L0 exp(t/tau) with tau~40h) (Zagorski et al, Science, 2017, DOI: 10.1126/science.aam5887), and some studies found an approximately linear growth (Cohen et al, Nat Commun, 2015, DOI: 10.1038/ncomms7709). In our model, implementation of exponential vs linear growth does not qualitatively alter the results. We have included this new data in Fig. 4A, A', B, and B' of the revised manuscript. This is consistent with the analysis of the model behaviour at different linear growth rates that we initially presented in Fig. 4 – these data show that the FP size scaling and its independence of Shh^{FP} were maintained across a broad range of growth rates, as well as in the condition of no growth.

Our motivation for not modeling cell divisions explicitly was based on several observations. 1) It is known that cell division in this tissue is spatially uniform and asynchronous (Kicheva et al, Science, 2014, DOI: 10.1126/science.1254927). 2) In our previous work (Bocanegra-Moreno et al, Nature Physics, 2023, DOI: 10.1038/s41567-023-01977-w; Guerrero et al, Development, 2019, DOI: 10.1242/dev.176297), we studied the dynamics of the apical surface of the neural tissue both experimentally and using a cell-based mechanical vertex model that included cell divisions. Our results indicated that cell rearrangements in this tissue are limited, hence cells retain their approximate relative positions as the tissue grows. 3) At early developmental stages (prior to 30h), the neural tissue is known to grow by symmetric cell divisions (e.g. Saade et al, Cell Reports, 2013, DOI: 10.1016/j.celrep.2013.06.038), in which Shh signaling and cell fates are thought to be inherited equally by daughter cells. Altogether, this growth process is well approximated by dilution and advection terms in continuum models, similar to what has been demonstrated in other epithelial tissues (Farhadifar et al, Current Biology, 2007, DOI: 10.1016/j.cub.2007.11.049; Averbukh et al, Development, 2014, DOI: 10.1242/dev.107011; Wartlick et al, Science, 2011, DOI: 10.1126/science.1200037). The expanding bin size in our models captures these effects.

To explicitly show that the number of bins in our model does not influence the results, we have now performed simulations with increased number of bins. As expected, the resulting FP size and Shh amplitude dynamics were unchanged in this scenario (Reviewer Fig. 1).

Nevertheless, the continuum deterministic nature of our model does not allow us to specifically consider possible stochastic effects introduced by cell division. We have previously shown that increasing the rate of cell division can increase the imprecision of gene expression boundaries in the neural epithelium (Bocanegra-Moreno et al, Nature Physics, 2023, DOI: 10.1038/s41567-023-01977-w). Therefore, to address the reviewer's concern and represent the possible imprecision of F specification introduced by cell divisions, we introduced noise terms within the gene expression network, in particular in the levels of F and N. As we expected, this resulted in imprecise F domain boundaries, with some positions within the N domain incorrectly expressing F and vice versa – this effect is similar to what we have observed in vertex model simulations with high division rate (Bocanegra-Moreno et al, Nature Physics, 2023, DOI: 10.1038/s41567-023-01977-w). The magnitude of the imprecision increased with the magnitude of noise. To determine whether the presence of this imprecision altered our main conclusions, we analyzed the sensitivity of the model with noise to $\kappa_{F\to S}$. Importantly, we found that the result was very similar to the scenario without noise – the model showed very weak dependence on $\kappa_{F\to S}$. This demonstrates that the insensitivity of the model of Shh production in the FP is a general feature that is robust to gene expression noise. These new simulation results are presented in Fig. S2B-E of the revised manuscript and are introduced in lines 236-238:

Furthermore, the robustness of the model to changes in $\kappa_{F\to S}$ values was also preserved in the presence of noise in gene expression (Fig. S2B-E).

The noise as a proxy for stochastic effects introduced by cell division is now introduced in Methods, lines 145-150:

As a proxy for stochastic effects resulting from cell division, we additionally simulate the deterministic system described in Eq. 1 with fluctuations in $[F]$ and $[N]$ as an Ornstein–Uhlenbeck process. We used the discretization following (30): $\eta(t+\Delta t) = \eta(t)\, exp(-\Delta t/\tau_\eta) + \sqrt{\sigma_\eta(1-exp(-2\,\Delta t/\tau_\eta)/\tau_\eta}$ $\alpha(t)$, where η stands for $[F]$ or $[N]$, $\alpha(t)$ is a Gaussian white noise with zero mean and unit variance, σ_η is the magnitude of noise, and τ_η is correlation time of noise. The noise is included in $[F]$ and $[N]$ only when the respective expression levels are higher than 1 a.u.. The results with noise are presented in SM.

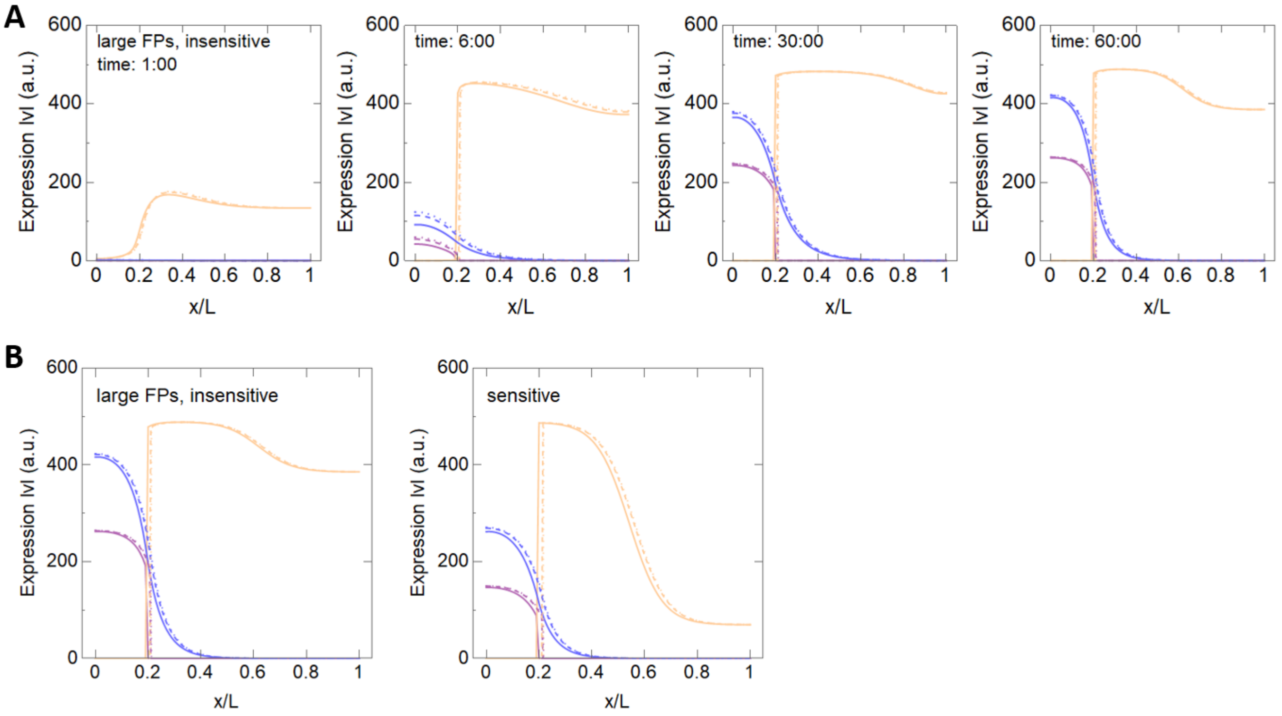

Reviewer Fig. 1. Spatiotemporal patterns with varied number of bins. A. Time course of spatial pattern for the FP domain (purple), neural progenitor domain (orange), and Shh (blue) for different numbers of bins: 100 (solid), 200 (dashed), 400 (dotted). **B.** Spatial pattern at the end of simulation (t=60h) for large FPs (l_{FP}/L_{end} = 20%) for insensitive (left) and sensitive (right) class of solutions. The results are shown for a randomly selected solution, n=1; we investigated n > 10 for different conditions and all exhibited very similar dynamics of FP size and Shh amplitude as the number of bins was increased from 100 to 200 and 400.

Furthermore, the effect of the notochord which produces the Shh that regulates growth of the floorplate in the initial stages is only indirectly implemented by a constant flux of Shh. I am wondering if there could be temporal variation in the flux of Shh produced by the notochord that is experienced by the floorplate as it grows. This could be intrinsic or arise from domain size changes.

This is an interesting question. In a separate ongoing project, we measured the mouse notochord size and shape during developmental time in effort to understand whether the growth of this organ could modulate the net production of Shh over time at a given position in the embryo. Surprisingly, we found that the notochord increases in length, but its cross-sectional area remains constant along most of the anterior-

posterior extent of the body axis and also over time (see Reviewer Fig. 2A below). This suggests that the notochord does not produce more Shh over simply by growth. To further confirm this, we quantified the Shh fluorescence intensity in the notochord in our wildtype embryos and found that it does not correlate with anterior-posterior position (Reviewer Fig. 2B), further confirming that Shh production in the notochord does not increase over time.

So far, the field has rather focused on the converse problem – a potential decrease in the Shh flux from the notochord over time. This is based on the observation that at early stages, the notochord is directly adjacent to the neural tube, while from E10 onwards (~50h at brachial region and in our simulations) a small gap progressively forms between the two tissues – this process is known as 'notochord regression' (Yu et al, Development, 2013, DOI: 10.1242/dev.090845); we briefly refer to this in the discussion on lines 560-563:

> *In mouse development, the notochord is in direct contact with the neural tube until approximately the 30 somite stage, while subsequently this contact is lost (14). It has been suggested that this may lead to an inability of the Shh derived from the notochord to continue spreading to the neural tube ((14, 15), also see (71)).*

The Yu et al study found that Shh^{FP} contributes to the correct maintenance of neural progenitor domains (Nkx2.2 and Olig2), as well as to gliogenesis, from the time point of notochord regression onwards. Based on this evidence, they suggested that Shh produced in the notochord may not reach the neural tube after regression. Although this has not been directly tested, in our manuscript we perform simulations in which we remove the notochord flux at different time points - this situation is similar to what would be expected if notochord regression led to cessation of the flux. These results are presented in Fig. 5E and F of the manuscript show that removal of the flux from 10h or 20h onwards (which is earlier than the suspect time point of notochord regression) has no effect on the floor plate size. These results are further confirmed experimentally in Fig. 7H, I – there we show that removal of all Shh input from E9.5 onwards has no effect on the floor plate.

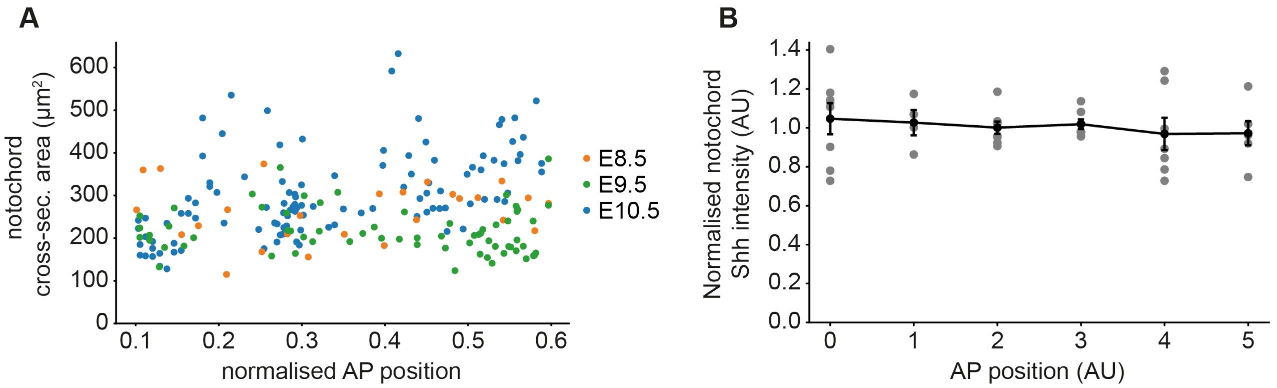

Reviewer Fig. 2. The size of the notochord and the levels of Shh remain stable across anterior-posterior (AP) position and developmental time. A. The cross-sectional area of the notochord measured in the embryo trunk, at distinct AP positions and developmental stages (indicated colors). The AP position is normalized so that 0 corresponds to the anterior and 1 to the posterior end of the notochord at all stages analyzed. **B.** Mean Shh intensity from immunostainings of wildtype sections collected at different positions along the AP axis at E10.5. Here, small values correspond to anterior brachial sections, while higher values correspond to posterior brachial sections. Grey dots – individual tissue sections, black dots – mean, error bars – SEM.

Reviewer #2:

The manuscript presents a computational model designed to account for the general regulatory principles that control the establishment and size of the floor plate in the vertebrate neural tube. The principles are reduced to the simplest possible form for the model, with Shh from the neurla tube inducing expression in the neural plate/tube of Shh and generic "floor plate" and "neural" factors, which are mutually antagonistic.

Through random search of parameter space, it is shown that appropriate behaviour (establishment of an appropriately sized floor plate on an appropriate timescale) is achieved for a large fraction of parameters. The model behaviour can be understood as falling into three categories, determined by the dependence of floor plate size/persistence on floor plate-derived Shh.

The model gives new insight into an old question of the relative roles of Shh from the notochord and floor plate, and provides experimental data to complement the modelling study. I think the model is appropriate and the modelling carried out well, and that this is a worthwhile contribution to the field.

I don't have any significant concerns, but there are a few points where some more methodological detail is needed.

We thank the reviewer for appreciating our contribution to the field as "worthwhile" and for their supportive and constructive feedback.

1. The model includes "dilution" resulting from tissue growth (referred to on p.5). Spatial bins are used to discretise the neural tissue, and these expand. However, it is not explained how this dilution is implemented. How do the differential equations change over time to reflect dilution?

Indeed, this needs further explanation, as we only mentioned that "both the dilution and advection effects resulting from growth are handled by the numerical integration scheme". The reviewer is right that in order to implement the dilution of $[F]$, $[N]$ and $[Shh]$ due to growth, Eq. 1 needs to be modified. This is achieved by adding an extra term "$-c\,\dot{L}/L$" to each equation in Eq. 1, where c is the respective concentration, and $\dot{L}$ is the rate of change of $L(t)$. In the reaction-diffusion equation, the diffusion is modified by rescaling from D_S to D_S/L^2. This follows the standard formulation of reaction-diffusion equations on growing domains (see for instance (Crampin et al, Bulletin of Mathematical Biology, 1999, DOI: 10.1006/bulm.1999.0131); now cited as (26) in the main text). The system is solved on a unit interval of $0 \leq \bar{x} \leq 1$, with coordinates in absolute units retained by $x = L\bar{x}$.

This scheme is valid for position-independent growth as is the case here. In particular for the linear growth condition, defined as $L(t) = L_0 + k_p t$, we obtain $\dot{L}/L = k_p/(L_0 + k_p t)$, and for the exponential growth, defined as $L = L_0 \exp(t/\tau)$, we have $\dot{L}/L = 1/\tau$, so for both conditions the relative rate of change of tissue length is position-independent.

We now clarify in the manuscript, lines 125-132:

> *The growth is implemented by introducing in Eq. 1 the new spatial variable $\bar{x}$ for which the domain size is constant (26). This introduces additional, concentration-lowering term $-c\,\dot{L}/L$ into each equation, where c is respective concentration of $[F]$, $[N]$ and $[Shh]$, and $\dot{L}$ is the rate of change of $L(t)$. We also use the diffusion constant rescaled to D_S/L^2. The equations are solved on the unit interval $0 \leq \bar{x} \leq 1$, divided into 100 spatial bins that mimic the discrete cellular structure of the tissue. The integration scheme uses first order finite differences of the spatial derivatives, whilst time steps are handled using the Heun's scheme, which is a second order method. The solution in absolute units is retained by $x = L\bar{x}$.*

2. Shh from the notochord is modelled either as a transient burst or as a constant flux at one end of the spatial domain. There are two issues here: (a) the first part of the "Results" section uses the transient burst

condition, but this is only stated after the results have been presented (half way down page 10, when the other condition is introduced). The conditions should be stated clearly at the start of the Results section;

We thank the reviewer for noting that this has been unclear. We have now modified the first paragraph of the Results section to include the information of the initial conditions used in the modeling results that follow, and we explicitly mention the transient burst condition, lines 208-209:

> *The gene regulatory network starts from an initial state with high N expression, no F expression and responds to an initial burst of Shh to activate F (Fig. 1C).*

(b) the boundary conditions are not stated (or I couldn't see them). I'm guessing zero flux at both ends (once the constant flux of Shh is turned off). These should be stated.

Thank you for noticing. Yes, it is zero flux at both ends, and then after the flux is switched on, only at the dorsal end (x=L). We state this in the Methods clearly indicating what are the initial and boundary conditions considered in the study, lines 137-144:

> *To represent and evaluate the temporal requirements for Shh secreted by the notochord (27–29), we consider different combinations of initial and boundary conditions: 1) a transient burst of Shh at position x = 0 within the tissue ($[S]_{init}$ = 100 a.u. at x = 0, and $[S]_{init}$ = 0 for $0 < x \leq L_{end}$) with double reflective boundary conditions $\left.\frac{\partial[Shh]}{\partial x}\right|_{x=0} = \left.\frac{\partial[Shh]}{\partial x}\right|_{x=L}$ = 0, 2) no Shh present in the tissue at t = 0, but a constant flux of Shh j_{Shh} through the ventral end of the neural tube $\left.\frac{\partial[Shh]}{\partial x}\right|_{x=0} = -j_{Shh}$, with the reflective boundary condition at the dorsal end $\left.\frac{\partial[Shh]}{\partial x}\right|_{x=L}$ = 0, 3) similar to the previous condition, but in which the flux of Shh, j_{Shh}, is abruptly removed at a specific time t_{off}. After the flux is removed, the boundary conditions are double reflective. Throughout the text and figures, we refer to $[S]_{init}$ also as S_{init}.*

3. It isn't clear to me why the study uses the two different Shh conditions. The constant flux approach seems more appropriate as a model of Shh from the notochord. It would be good to comment on this a bit more, and explain to what extent the outcomes depend on the details of this condition.

We agree with the reviewer that the constant flux is a more realistic representation of the notochord. Nevertheless, the transient burst condition was motivated by previous studies that indicated that the notochord is critically important for pattern formation of the dorsal neural progenitor domains early on (e.g. Chamberlain et al, Development, 2008, DOI: 10.1242/dev.013086; Yu et al, Development, 2013, DOI: 10.1242/dev.090845). One of our goals was therefore to understand how the system dynamics depends on these two conditions of Shh present only initially as opposed to constantly incoming Shh. To our surprise, we found that for specific values of flux, both conditions yield quantitatively and qualitatively similar outcome with respect to floor plate size. This data is presented in Fig. 5B, in which the dashed lines indicate the flux values that yield the same relative FP size as the burst condition for two subsets of solutions, as well as in Fig. 5D.

In the revised version of the manuscript, we performed further simulations to illustrate how FP size depends on the magnitude of the initial burst. We found that this dependence is logarithmic, which is expected from a French flag type model in which the target gene domain boundary is specified at a given concentration threshold. This new data is presented in Fig. 5A. We further revised Fig. 5B and C, as well as the text in line 288-295:

> *While these observations demonstrate the importance of the initial Shh signalling levels for setting the FP size, in the embryo, Shh is continuously produced by the notochord, rather than as a short burst, which raises the question of how the dynamics is affected by continuous production of Shh. To simulate the effect of the notochord, we compared our default model with an initial pulse of Shh to a model with a constant*

flux of Shh, representing the notochord. We found that for a specific magnitude of flux, the model produced a similar relative FP size as in the model with a pulse (Fig. 5B). Similar to the magnitude of S_{init} (Fig. 5A), increasing flux lead to a logarithmic increase in the relative FP size (Fig. 5B). This was the case for both sensitive and insensitive classes of solutions (Fig. 5B, C).

The introduced changes illustrate now more clearly that j_{Shh} is related to FP size via the same logarithimic relationship. The similarity in FP formation between the burst and flux condition simulations supports our main conclusion that the Shh input is important for establishing the FP only at early time points.

However, another key conclusion that arises from the comparison between the burst and flux conditions is that they differ with respect to the Shh amplitude at late time points. In the flux condition, the Shh amplitude is increased compared to the burst condition by a factor that depends on the flux magnitude. To enable the reader to make this comparison easily, we now revised Fig. 6E, F to plot the burst and the corresponding flux condition on the same plot. Furthermore, also in Fig. 6E, F, the same effect can be appreciated by following abrupt cessation of the flux that resembles a burst condition and causes a very similar difference relative to the continuous flux condition.

To improve the clarity of our manuscript, in addition to the figure modifications, we have revised the relevant paragraph starting on line 373, including the fragment in which we explain more clearly the rationale behind the comparisons of the flux and burst conditions, lines 373-385:

Altogether, our analysis suggests that the floor plate and the Shh it produces have an essential contribution to the Shh amplitude dynamics. Nevertheless, it is possible that Shh derived from the notochord also contributes to the Shh amplitude. To investigate this, we first asked how the Shh gradient amplitude behaves in a model where Shh is continuously supplied by the notochord (j_{Shh}) but is not produced in the FP. In this case, the Shh amplitude was linearly related to j_{Shh}, $A_{Shh} \sim j_{Shh}$, as expected from a model of gradient formation by diffusion, degradation and localized production (39) and was lower compared to the condition with Shh^{FP} production (Fig. S7C). This supports the conclusion that the presence of the FP amplifies the Shh amplitude. We then analysed the converse scenario, in which Shh is produced in the FP ($\kappa_{F \to S}$ > 0) but not in the notochord. To this end, we compared the model with continuous flux from the notochord j_{Shh} to the conditions with initial burst of Shh or with j_{Shh} abruptly removed at 10h, which have no continuous notochord contribution. We found that if the Shh flux is abruptly stopped or Shh is provided only as an initial burst, the A_{Shh} does not reach the same extent as with continuous j_{Shh} (Fig. 6D-F), however, the difference between the conditions depends on the magnitude of the flux.

Overall our simulation results show that the level of Shh in the tissue is predicted to depend on the combination of FP-derived Shh and notochord-derived Shh. However, our experimental results indicate that the notochord-derived Shh contributes little to the Shh amplitude at late stages. We conclude this by comparing the Shh gradient in the condition where FP-derived Shh is removed but notochord Shh is unperturbed (Fig. 7D) to the conditions where both FP and notochord Shh are removed at early and late stages (Fig. 7G, and J, respectively). In all of these cases, the gradient shape is similar. This data supports our conclusion that the contribution of notochord-derived Shh to the gradient is small relative to the contribution from the FP-derived Shh. Thus, based on our experimental data, the actual value and contribution of the flux is low, e.g. more similar to Fig. 6F than to 6E.

4. N and F regions are said to be determined by simple inequalities N>F and F>N. There is only one figure showing the actual profiles (Fig 1C) and this shows clear "mutual exclusion" (effectivelty no coexistence of N and F). Is this always the case for "acceptable" parameter sets, or do some have regions where both N and F are expressed at intermediate levels? I expect not given the exponent of 3 ("strong" bistability), but it would be good to know.

For the successful parameter sets, F and N were co-expressed only transiently in the first ~20h of the simulation. For most sets, maximum F expression in the floor plate region was reached within 10h, while N

was completely repressed by 20h. We now show the mean expression levels over time in the floor plate in Fig. 1C of the revised manuscript, and in addition we provide a more detailed view of the results in Reviewer Fig. 3 below where we show 100 randomly chosen example parameter sets. From these plots, it can be seen that after ~20h, there were no cases with co-expression of both N and F.

Interestingly, it has been experimentally shown that during the first ~24h of floor plate induction, the floor plate marker FoxA2 and Nkx2.2, which marks the adjacent p3 domain, are co-expressed within the same cells, while at later stages this is no longer the case and the two domains are mutually exclusive (e.g. Jeong & McMahon, Development, 2005, DOI: 10.1242/dev.01566; Mansour et al, Development, 2014, DOI: 10.1242/dev.104372; Lek et al, Development, 2010, DOI: 10.1242/dev.054288). Thus, although the gene regulatory network in the real system is more complex than the simplified network that we model in this study (as we point out in the Discussion), our model captures well the observed sharp separation of the FP and the adjacent domain at late developmental stages.

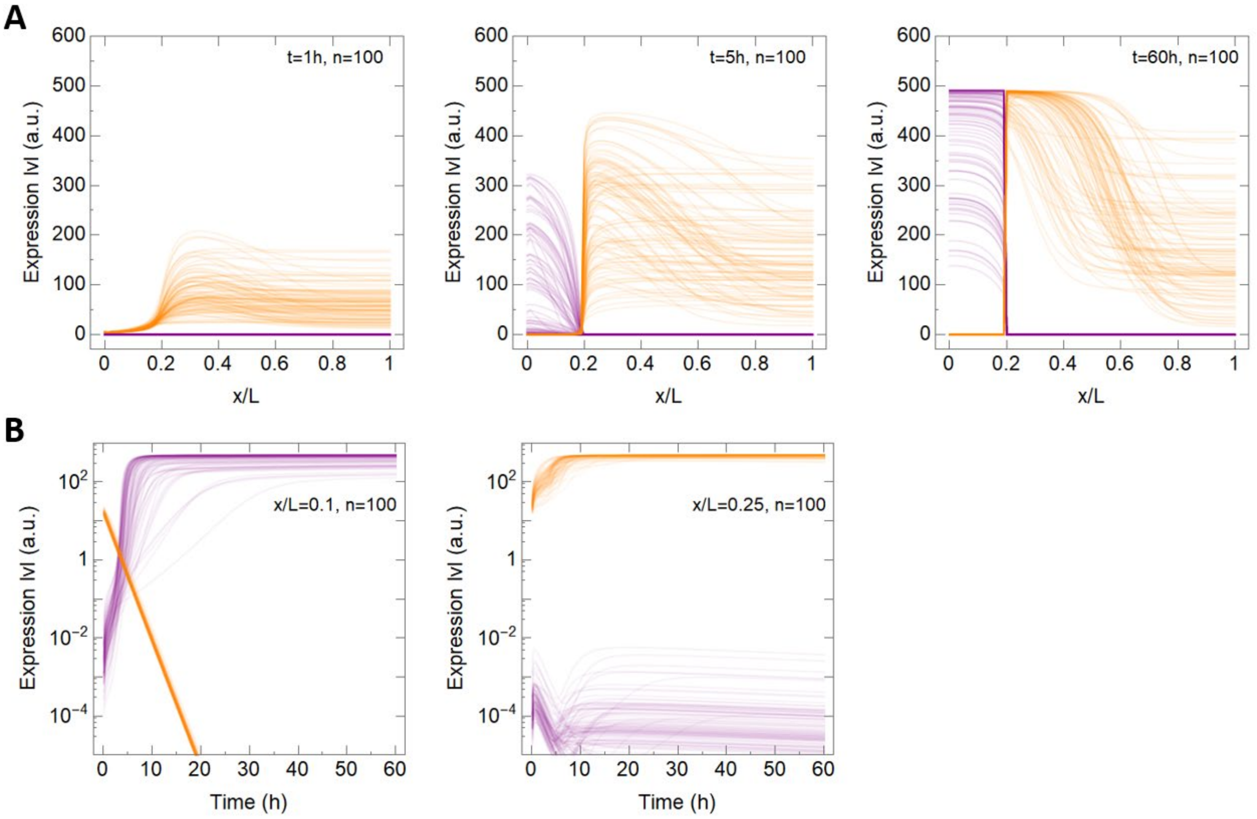

Reviewer Fig. 3. Individual gene expression profiles of F and N. A. Profiles of F and N for n=100 randomly selected Shh-insensitive solutions resulting in a large FP (l_{FP}/L_{end}= 20%). Spatial profiles at 1h, 5h, and 60h, for F (purple) and N (orange) are shown. **B.** Temporal profiles of the same set of successful solutions at x/L = 0.1 and x/L = 0.25, for F (purple) and N (orange).

5. As I understand it, the key principle of the model is that N and F are mutually antagonistic, and that N--F is bistable for a wide range of parameters (certainly with Hill exponents of 3). Shh contributes to setting the threshold, so it is possible to find parameter regimes where this is an important contribution and regions where it is not. Would it be possible to give a more generic description of what the model is doing (in a dynamical systems type way) that doesn't fixate on the neural tube? It's a bistable switch with a regulating input that is spatially distributed. It might be interesting to compare and contrast it to other instances of that: e.g. regionalisation in the neural tube; gap gene domain establishment in the Drosophila blastoderm.

Thank you for this insightful suggestion. Many of the patterning motifs that have been studied in neural tube and Drosophila gap gene patterning contain cross-repressive interactions, but not positive feedback. A

distinct feature of our model is that it contains a positive feedback motif, however, as we show – this positive feedback is only relevant for the activity of the network in certain parameter regimes, which are actually not relevant to the in vivo situation. In other parameter regimes, the positive feedback does not contribute to patterning and is therefore more similar to DV patterning networks in the neural tube (Balaskas et al, Cell, 2012, DOI: 10.1016/j.cell.2011.10.047; Zagorski et al, Science, 2017, DOI: 10.1126/science.aam5887).

In the work (Majka et al, Physical Review Letters, 2023, DOI: 10.1103/PhysRevLett.130.098402), we showed that in the case of a source domain that produces a morphogen that self-activates its own production, the condition for the formation of a stable domain requires fine-tuning of parameters, and generic behaviours are an expansion of the domain with constant velocity (the tissue does not grow) or a collapse/decrease in the size of the domain when degradation overcomes the Shh auto-activation. In our current work, in terms of a generic mechanism, the expansion of FP predicted by the model from Majka et al. is blocked by N, which acts as a wall for F expression.

We have now included the following paragraph in the Discussion, lines 496-511, to address the reviewer's point:

> *In general terms, the patterning system that we identified corresponds to a bistable switch coupled to positive feedback, driven by spatially distributed morphogen input and uniform basal activators. Similar networks operate tissue patterning of other systems (56, 57). Studies on DV patterning of the neural tube (16) or gap gene patterning in the Drosophila embryo (58) have shown that the exact positions of target gene domain boundaries depend on the integrated input of multiple regulators, and are not simply proportional to the morphogen concentration. In these systems, the network interactions lead to the locking of the system in attractor states that correspond to defined cell fates (59). This gives rise to complete or partial independence of the cell fates from the initial morphogen input (e.g. hysteresis) (16, 18, 60–62). In our model, the sensitivity of the pattern to morphogen input depends on the network parameters. In the 'sensitive' parameter regime, morphogen input matters for FP formation continuously by engaging the positive feedback loop, while in the 'insensitive' regime, Shh input is required only transiently and FP formation occurs independently of the positive feedback. While the sensitive networks correlate with weaker repressive interactions, the insensitive class networks require only weak activation of FP identity by Shh from the notochord and strong repressive interactions, as well as a larger contribution from uniform basal activators. This 'insensitive' class of networks, which we show is the one relevant to the in vivo situation, has similar properties to other known bistable patterning systems (18, 63).*

Reviewer #3:

In this manuscript, Ho et. al., investigated the biophysical mechanisms underlying formation of morphogen source in the ventral neural tube. Specifically, the authors investigated the contribution of different factors to the size of the floor plate and the Shh morphogen gradient dynamics. By performing a data-constrained computational parameter screening, they tested several potential biophysical mechanisms and found that only one mechanism fitting to experimental data. According to the simulation-favored mechanism, the floor plate is specified by the Shh produced from the notochord and a regulatory network responding to it in the neural tube and floor plate. After it is specified, the floor plate becomes mostly insensitive to Shh, and its size increases only due to tissue growth. This two-step regulation results in scaling of Shh amplitude with tissue growth. Overall, I liked the manuscript. I suggest the following improvements:

We thank the reviewer for their positive assessment of our manuscript and their constructive feedback.

1) What is the significance of an amplitude increase in the floor plate? If the gradient encodes information not at absolute levels but let's say at its spatial fold change, then an amplitude increase might not matter for growth control and/or maintenance of the floor plate identity. Experimental findings in this paper also suggests that floor plate size can still accurately forms despite 95% decrease in the Shh amplitude. Similarly, in ShhCreERT2/+ heterozygous embryos, while the Shh gradient amplitude was reduced, the floor plate appeared normal. This major issue has not been detailly and adequately discussed in this manuscript. I think it is important to communicate the significance of this work to outsiders from the field and to make a broader impact in the larger developmental biology field.

We thank the reviewer for pointing out the need for further clarification. Indeed, as the reviewer notes, our data indicate that the continuous increase of the Shh gradient amplitude does not matter for the maintenance of floor plate identity. Prior studies suggest that the Shh amplitude increase is important for the specification of the neural progenitor domains, e.g. Nkx2.2 and Olig2. For instance in (Yu et al, Development, 2013, DOI: 10.1242/dev.090845), they show that the number of cells in the Nkx2.2 and Olig2 domains are reduced when Shh production is abolished in the floor plate, however, similar to our data, they found that the size of the floor plate is not affected.

Our model provides an explanation for this. Rather than the increase in amplitude over time, what matters for the specification of the floor plate size is the initial level of Shh. To support this, in Fig. 4A and C of the manuscript, we show that a short initial burst of Shh without Shh^{FP} production (Fig. 4C) leads to the same FP size as with Shh^{FP} production (Fig. 4A), which indicates that Shh^{FP}, which drives the amplitude increase, is not important for FP size.

To further demonstrate that the FP size depends on the magnitude of the initial burst of Shh, we have now included additional simulation results and revised the text. In our new simulation results, included in Fig. 5A of the revised manuscript, we show that the FP size depends logarithmically on S_{init}. These new data are also consistent with the data we report in Fig. 5B, which shows that the FP size increases logarithmically with increasing flux j_{Shh} (which is present from t = 0h). To make this clearer, we now provide the data both in linear and log scale and we extended the figure legend accordingly (see Fig. 5B, C). The logarithmic relationship is expected if an exponential gradient defines target gene positions at a specific concentration threshold (reviewed in (Wartlick et al, Cold Spring Harb Perspect Biol, 2009, DOI: 10.1101/cshperspect.a001255). In this case, $x^* = \lambda \ln(c_0/c^*)$, where x^* is the target gene boundary position, λ the decay length, c_0 the gradient amplitude and c^* the threshold concentration. As we explain in the revised text, in the model the level of S_{init} is proportional to the amplitude of the initial transient gradient, and similarly the flux j_0 (j_{Shh} in our convention) is proportional to the gradient amplitude via $j_0 = c_0 \sqrt{D\,k}$ (Kicheva et al, Science, 2007, DOI: 10.1126/science.1135774), where D is the diffusion constant and k is the degradation rate. The logarithmic relationship means that the expected change in x^* (FP size) in response to a 2-fold change in the initial gradient amplitude may be anywhere in the range between 1 and 2-fold,

depending on c^* and λ, i.e. it is possible that a 2-fold reduction in the initial amplitude will produce no detectable change in FP size.

We have also revised and restructured the experimental validation part of the manuscript to better explain how the simulation results are supported by the experimental data that we initially presented in the manuscript, as well as by new results that we added.

The first approach that we took was to change the timing of Shh deletion and assess how this affects FP size. Deletion of Shh at late time points, after FP formation, is presented in Fig. 7B, C (only FP), and H, I (both notochord and FP). In both of these cases, the amplitude decreases by 91% and 87% respectively, but the FP remains intact. By contrast, a strong decrease in Shh production at early time points lead to a very strong decrease in floor plate formation (Fig. 7E, F). This supports our interpretation that only the initial Shh levels are important for FP formation, while the Shh levels at later time points are not important.

In the second approach, we varied the levels of Shh production, starting at early times. For this, we used Shh heterozygous embryos, which have constitutive reduced Shh production in the notochord and floor plate due to the loss of one copy of the gene. In these embryos, the average Shh amplitude is reduced to ~40% of wildtype. We additionally performed a new experiment in which we generated $Shh^{CreERT2/Flox}$ embryos with a partial reduction in Shh production at early times by injecting them with a low tamoxifen dose. In these embryos, the final amplitude of Shh was reduced to approximately 25% of wildtype. We took further advantage of the variability in the data to analyze the relationship between final Shh amplitude and FP size in individual tissue sections of wildtype embryos, heterozygous and homozygous mutants. These new results, reported in revised Fig. 8C and introduced in the paragraph starting at line 414, show an approximately logarithmic relationship between FP size and the final Shh amplitude across conditions.

In order to relate these experimental results to the model, we performed simulations in which the production rate of Shh was reduced in both the floor plate (α_S) and in the notochord (j_{Shh}). Remarkably, these simulations yielded a curve that was in an excellent agreement with the experimental data as shown in Fig. 8C of the revised manuscript. This analysis shows the reduction in Shh amplitude in the heterozygous embryos is consistent with the lack of appreciable reduction in FP size. More generally, this reveals that the FP size is highly sensitive to Shh levels for low initial production rates, but is relatively robust when the initial production rate is high.

We now describe this analysis in the revised text, lines 414-434:

> *The second prediction of the model is that the floor plate size depends on the initial levels of Shh (Fig. 5A). To test this, we took advantage of experimental conditions in which the production of Shh from the notochord and floor plate was affected to varying extents from the onset of neural tube formation. One such condition is Shh heterozygous embryos, which have only one functional copy of the Shh gene – we found that in these embryos the Shh levels in the notochord (at E8.5) and the gradient amplitude (at E10.5) were reduced compared to wildtype controls (Fig. 8A, B, S8A, B). To reduce Shh production even further, we generated $Shh^{CreERT2/Flox}$ embryos induced with a low dose of tamoxifen. In this condition, only a fraction of notochord and floor plate cells contain one functional Shh copy, whereas the other cells do not express any Shh. This reduces the Shh amplitude to levels between the heterozygous mutants and the high dose-injected mutants (Fig. 8A, B). Together, the set of the wildtype, heterozygous and homozygous conditional mutants had a broad range of Shh amplitudes. By plotting the Shh amplitude and corresponding FP size in each tissue section across these conditions, we found that floor plate size was non-linearly related to the Shh amplitude at E10.5 (Fig. 8C), reminiscent of the logarithmic dependence of l_{FP} on S_{init} and j_{Shh} (Fig. 5A, B). Strikingly, simulation of the relationship between the final amplitude and final floor plate size for conditions with decreased Shh production (α_S and j_{Shh}) was in an excellent agreement with the experimental data (Fig. 8C). This result supports the validity of the model and shows that the floor plate is dependent on the levels of Shh production from the earliest developmental stages. Furthermore, this analysis shows that the relationship between amplitude and FP size in embryos with different levels of Shh production is non-linear: FP size sharply increases with A_{Shh} at low production values and is less sensitive*

to changes in the amplitude at high values. This non-linear relationship provides an explanation for the relatively unperturbed floor plate formation that is observed in Shh heterozygous embryos (Fig. 8A, D).

Furthermore, we included an additional paragraph in the Discussion summarizing our new theoretical and experimental result and explicitly addressing the phenotype of Shh heterozygous embryos, lines 512-522:

> *Although the FP depends only transiently on activation by Shh from the notochord, this initial activation influences the FP size. Our experimental and theoretical analysis shows that the FP size depends non-linearly on the levels of Shh production at early times, being highly sensitive to low levels and relatively robust at high levels of Shh. This non-linearity arises as a collective result of the exponential nature of the gradient at its early impact on FP specification and the independence of the FP from Shh input at late times, which arises as a result of the gene regulatory network dynamics. This feature of the system explains our counterintuitive observation that Shh heterozygous embryos, in which the Shh amplitude is approximately halved, have no detectable phenotype in floor plate formation. Such robustness of the FP size to Shh levels may also potentially be achieved by additional non-linear dependencies of the gene regulatory interactions on Shh and it would be interesting to explore this possibility in future studies, taking into account more detailed representations of the regulatory network.*

In this paragraph, we add that although our analysis provides an explanation for the observations, we cannot exclude a potential role for additional interactions that we have not considered in the model.

We thank the reviewer for this question, since the additional theoretical and experimental results, as well as the corresponding revisions to the text have helped to strengthen our manuscript and increase the clarity of our arguments.

2) The initial simulations showed that FP size is least sensitive to the Shh production in the FP. This suggests that the FP may form in a manner that is largely independent of the Shh that it produces. I wonder whether this conclusion depends on the choice of diffusion coefficient value, which is fixed and not varied in the simulations (or the Shh flux into the tissue). Please explore these possibilities and report the outcome (positive or negative).

As requested, we have addressed this question by analyzing the outcome of simulations with varied diffusion coefficient. We found that FP size decreased as D_S was decreased, and vice versa, it increased with increasing D_S. This was the case for all Shh-insensitive, and most partly-sensitive and sensitive classes of solutions. In a few cases of the sensitive class, higher D_S lead to the loss of FP formation, because the fast diffusion lowers the Shh concentration close to the source below the levels necessary for activating F. However, most importantly, the sensitivity of the model to Shh production in the FP is not affected by the value of D_S (new Fig. S2A). Thus, even for large values of D_S, the insensitive solutions remain insensitive to Shh^{FP}.

We report the impact of varied flux in the tissue in Fig. 5B, C. These figure show that the FP size depends on the Shh flux. This is the case for both sensitive and insensitive solutions. This result is consistent with the idea that the initial level of Shh, rather than its continuous production in the FP, sets the FP size. We further investigated whether the sensitivity of the FP size to the Shh production in the FP is similar for different levels of flux, but found it to be not affected by varied flux (Reviewer Fig. 4). Altogether, these results demonstrate that the existence of the insensitive class of solutions is independent of the choice of D_S or j_{Shh} in our model.

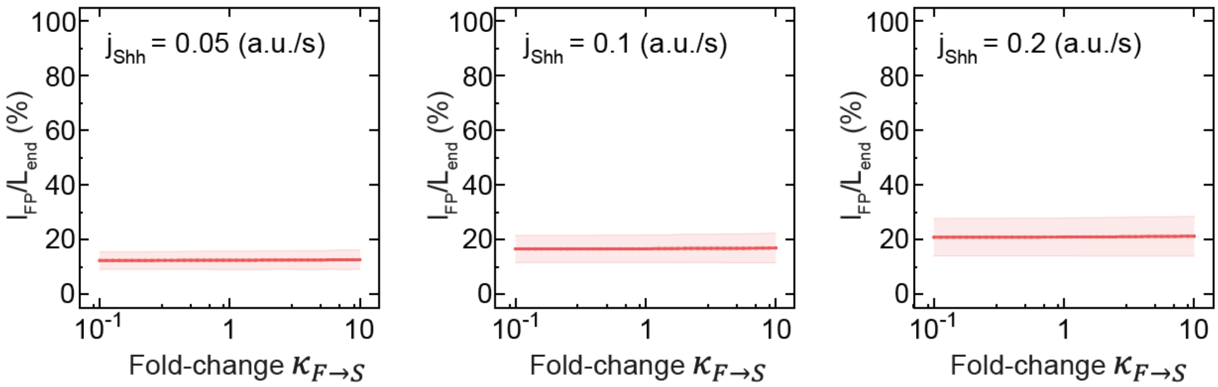

Reviewer Fig. 4. FP size sensitivity to perturbations of Shh production by floor plate. Mean relative FP size at the end of the simulation upon perturbation of $\kappa_{F\rightarrow S}$ for a subset of 50 randomly selected insensitive solutions resulting in large FPs (l_{FP}/L_{end}= 20%). The flux is varied with j_{Shh} = 0.05 a.u./s (left), j_{Shh} = 0.1 a.u./s (mid), and j_{Shh} = 0.2 a.u./s (right). The parameters are modified in 40 equally distributed logarithmic steps from 0.1 to 10-fold of their value. The shaded regions are SE.

3) The authors report that, in the insensitive class, FP formation results from strong basal uniform activation and repression by Shh-dependent repressors, with weak initial activation by Shh and with no activation by FP-derived Shh. Would not this result mean that the notochord-derived Shh is also almost irrelevant for FP formation? Please discuss this issue in the manuscript, if necessary, provide additional simulations and/or data.

We thank the reviewer for pointing out that this has been unclear. The initial activation of the FP by Shh is essential for FP formation. In the embryo, this initial Shh is provided by the notochord. Therefore, the notochord is of critical importance for FP specification early on. Our experimental data (Fig. 7E, F) show that if Shh is deleted from the notochord at early time points, then FP formation does not occur – this result is also consistent with previous findings by other researchers (Placzek et al, Development, 1993, DOI: 10.1242/dev.117.1.205; Ribes et al, Genes Dev, 2010, DOI: 10.1101/gad.559910). With respect to the later time points, after the FP domain is established (corresponding to the period after ~10h in our simulations), our simulations show that the Shh flux (which models notochord-derived Shh) does not influence FP size. If this flux is removed at 10h or 20h after the start of the simulation, there is no change in the FP size (Fig. 5E). This is also supported by our experimental data. In Fig. 7H, I, we show that if all Shh input is removed at a late time point, the FP size is unchanged.

To clarify this further, we have rephrased the text, in several places explicitly stating that the flux j_{Shh} in our model represents Shh coming from the notochord. Furthermore, in the lines 307-309, we now explicitly state that:

> *This supports the idea that any changes in the Shh flux from the notochord contribute to FP formation only early on, but that the FP does not respond to the continuous flux of Shh after this time point.*

We also added the following sentence to the Discussion, lines 474-475:

> *During this second phase, Shh input from the notochord and FP is dispensable for FP formation.*

4) Please explain why degradation rate of N is more influential than that of F in determining the time of FP specification. If necessary, perform new simulations.

In Fig. 4E we showed the time of FP specification decays with increasing degradation rate of N. For comparison, we now provide the dependence on the degradation rate of F. Indeed, T_{FP} changes less in response to γ_F than γ_N (new Fig. S4B, C). A possible intuitive explanation of this effect stems from the initial

conditions in the system. Initially, N is expressed everywhere, while F is zero. The critical factors to establish an F domain starting from that state are to repress or destabilize N, which is directly dependent on gamma N, and activate F, which depends both on the activation of F as well as on γ_F.

It is known that in non-linear systems, the time scales are not purely driven by degradation rates, but also by the Hill coefficients of activation and repression functions (Polynikis et al, Journal of Theoretical Biology, 2009, DOI: 10.1016/j.jtbi.2009.07.040; Gonze & Abou-Jaoudé, PLoS One, 2013, DOI: 10.1371/journal.pone.0069573) [now cited as (37, 38) in the main text]. The lack of sensitivity to γ_N is therefore possibly resulting from a buffering effect of some of the other interactions. Our data in Fig. S4A (showing the relationship between T_{FP} and parameter values, indicates that the range of parameter values corresponding to a given T_{FP} is in most cases very large, which is consistent with a complex multiparameter dependence of T_{FP}.

We have now revised the manuscript to better explain this point. We state now, lines 278-283

> *We found that there is no obvious correlation between the time it takes for the relative FP size to become constant (T_{FP}) and most individual parameters in the computational screen (Fig. S4A), consistent with observations that in non-linear systems, time scales may be determined by a set of parameters (37, 38). Nevertheless, we found that the time it takes for the relative FP size to become constant (T_{FP}) was most prominently influenced by the degradation rate of the reacting species, notably γ_N (Fig. 4E), and to a lesser extent γ_F (Fig. S4B, C).*

We thank the reviewer for this question, which helped us to improve and clarify our results.

5) The authors report that the growth of the floor plate, together with continuous Shh flux from the notochord, contribute to the increasing Shh gradient amplitude over time. Why does the Shh amplitude increase over time? Why it does not quickly reach to a steady state with the balance of production and degradation terms? The degradation rate might control that timescale. But is it so slow that the amplitude does not quickly reach a steady state?

The amplitude increases over time, because the floor plate grows and this leads to increasing the net production of Shh over time. Because growth is continuous, the production continuously increases and the amplitude does not reach a steady state. Consistent with this explanation, in the case when the tissue does not grow (growth rate = 0), the amplitude reaches a constant value in less than 10h and is maintained at that value. This result is shown if Fig. 6B. There is one exception to this – in the limit of very high growth rate (growth rate = 50 μm/h). In this case, the FP grows to a size beyond which any molecules that are produced are degraded before they reach the source boundary, therefore, further increase in the FP size does not contribute to further increasing the flux.

To clarify this point, we rephrased the text starting on line 356 as follows, lines 356-372:

> *In contrast to the decay length, the Shh amplitude increased with increasing growth rate (Fig. 6B). Furthermore, the temporal changes in amplitude depended on the growth rate. Crucially, in the absence of growth, the amplitude reached saturation levels in less than 10 h (Fig. 6B), indicating that growth is essential for the Shh amplitude to increase after this time point. For low and intermediate growth rates, the Shh amplitude increased linearly over time between 10 h and 60 h (Fig. 6B), as well as with respect to the growing tissue length L (Fig. S7A), indicating that the amplitude of Shh scales with the tissue size after 10 h.*
>
> *This suggests that tissue growth leads to an expansion of the FP size, which in turn leads to an increase of the overall Shh production, the net Shh flux into the target tissue and ultimately to an increase in the Shh amplitude over time (Fig. 6B). The size of the morphogen source, however, is expected to be linearly related to net morphogen flux through the source boundary and to the gradient amplitude only within a certain range. At large source sizes, newly produced molecules are degraded before they spread to the source*

boundary, hence further increase in the source size will not increase the flux (40, 41). Consistent with this, our simulations indicate that at an unrealistically high growth rate of k_p = 50 µm/h where the absolute FP size rapidly becomes large, the amplitude of Shh saturates over time (Fig. 6B). Similarly, in the complete set of successful solutions in the screen, we found that the Shh amplitude is on average linearly related to FP size for small FP sizes (< ~60 µm), and saturates for large FP sizes (Fig. S7B).

The authors separately reports that Shh is turned over on a time scale of a day or smaller than a day. Is there any experimental support for this rate? Such a result would increase the significance of this manuscript.

We thank the reviewer for this comment. In a recent preprint by the Briscoe lab (Benzinger et al, 2024 bioRxiv, https://doi.org/10.1101/2024.06.11.598403) [cited now as (25) in the main text], they measured a Shh degradation rate in neural progenitor cells of 0.22 ± 0.05 10^{-3} s = 0.79 ± 0.18 h^{-1}, which corresponds to a half-life of 52.5 minutes ($t1/2 = \ln 2 * \gamma_S^{-1}$). In our simulations, we have γ_S= 0.72 h^{-1} => t1/2= 58 min, which is remarkably close to the experimental measurement. Furthermore, they report a measurement of D =0.14 +/- 0.03 µm^2/s, which is also very close to the value of D_S = 0.11 µm^2/s which we used in our simulations. Thus, these exciting new results are consistent with our analysis and we now cite this reference in the text in line 114, Methods, lines 112-114:

To reduce the number of free parameters, we set the production rates α to 0.1 h^{-1}, degradation rates γ to 0.72 h^{-1} (= $2\cdot10^{-4}$ s^{-1}), and D_S = 0.11 µm^2 s^{-1} – these values are of similar order to the values measured for other morphogens (reviewed in (21)) and to inferred values for Shh in other studies (22–25).

Furthermore, we rephrased and restructured the paragraph that the reviewer is referring to, in order to explicitly refer to the experimental measurements, lines 435-442:

A third prediction of the model arises from the short (~1h) half-life of Shh, which has been experimentally measured (25) and is implemented in the model, which implies that the Shh gradient is continuously and rapidly turned over. This implies that Shh production is continuously required to increase the Shh gradient amplitude over time. To test this, we compared the Shh gradient shape in the $Shh^{CreERT2/Flox}$ conditional mutants, in which all Shh production was eliminated at early or late time points (Fig. 7A). As expected, deletion of Shh at both time points resulted in a severe reduction (by 91% and 87%, respectively) in the Shh gradient amplitude (Fig. 7G, J). Crucially, this result confirms that new production of Shh is continuously required to maintain the gradient amplitude.

6) The authors report that FP formation requires only initial Shh input from the notochord and is not sensitive to loss of Shh signalling at later stages. But they also provide experimental data showing that significant reduction of this notochord-contributed Shh dosage does not impair FP formation. Please discuss this matter detailly. I wonder whether FP formation depends on not absolute levels of Shh but to another feature of the Shh gradient. If the authors have any new data to address this question, that will dramatically increase the significance of this manuscript.

We have now addressed the phenotype of the Shh heterozygous embryos in detail in our response to question 1 above. We have presented new experimental data (Fig. 8), as well as theoretical analysis, showing that the initial Shh level is logarithmically related to FP size (revised Fig. 5A). This feature of the model arises because F formation depends on an absolute level of Shh at the initial time point and subsequently becomes independent of Shh – thus these data support our model. We have now explained this in detail also in the text and have added an additional paragraph in the discussion, lines 512-522 (the fragmented is cited in question 1 above). We thank the reviewer for bringing up this point, as we already highlighted in our reply to question 1, the additional theoretical and experimental results together with corresponding revisions have helped to strengthen our manuscript and increase the clarity of our arguments.
